# University of California

**Published:** 1886-02

**Authors:** 


### UNIVERSITY OF CALIFORNIA.

The fourth annual commencement exercises of the College of Dentistry, of the University of California, were held in connection with those of the Medical Department, at the Grand Opera House, San Francisco, on Tuesday, November 10th, 1885, at 2 o'clock.

Address on behalf of the dental department was delivered by Prof. Maurice J. Sullivan, D. D. S.

The degree of Doctor of Dental Surgery was conferred upon the following graduates :

Harry Sylvester Bettis, George Botsford, Daniel Barratt Cate, Nathaniel Thomas Coulson, George Ihnier Drucker, William Ellis Fitzpatrick, Walter Robert Henderson, John Adams Douglass Hutton, Franklin Pancoast, Charles Theodore Rodolph, Frederick Judson Saxe, A. M., Joseph Schneider, Henry Sylvester, Jr.

